# An expanded database of high-resolution MS/MS spectra for lichen-derived natural products

**DOI:** 10.1038/s41597-025-04488-w

**Published:** 2025-02-12

**Authors:** Joe Bracegirdle, John A. Elix, Udayangani Mawalagedera, Yit-Heng Chooi, Cécile Gueidan

**Affiliations:** 1 School of Molecular Sciences, The University of Western Australia, Perth, WA 6009 Australia; 2 Research School of Chemistry, Australian National University, Canberra, ACT 2601 Australia; 3 Australian National Insect Collection, National Research Collections Australia, NCMI, CSIRO, Canberra, ACT 2601 Australia; 4 Australian National Herbarium, National Research Collections Australia, NCMI, CSIRO, Canberra, ACT 2601 Australia; 5 Centre for Australian National Biodiversity Research (CANBR), Canberra, 2601 ACT Australia

**Keywords:** Metabolomics, Taxonomy, Mass spectrometry

## Abstract

The history of lichen compound identification has long relied on techniques such as spot tests and TLC, which have been surpassed in sensitivity and accuracy by modern metabolomic techniques such as high-resolution MS/MS. In 2019, Olivier-Jimenez *et al*. released the Lichen DataBase (LDB), a library containing the Q-TOF MS/MS spectra of 251 metabolites on the MetaboLights and GNPS platforms, that has been widely used for the identification of lichen-derived unknowns. To increase the compound coverage, we have generated the Orbitrap MS/MS spectra of a further 534 lichen-derived compounds from the metabolite library of Jack Elix, housed at the CANB herbarium (Canberra, Australia). This included 399 unique metabolites that are not in the LDB, bringing the total number combined to 650. Technical validation was achieved by investigating the compounds in three Australian lichen extracts using the Library Search and Molecular Networking tools on the GNPS platform. This update provides a much larger database for lichen compound identification, which we envisage will allow refining the lichen chemotaxonomy framework and contribute to compound discovery.

## Background & Summary

Lichens are mutualistic associations between fungi (mostly ascomycetes) and microalgae (mostly trebouxiophycean algae and/or cyanobacteria). With an estimate of up to 20,000 species worldwide^1,2^, they are present in most terrestrial ecosystems, colonizing a large range of natural (eg, rock, bark, leaves, soil) and artificial (eg, concrete, tiles, asphalt) substrates. Relying on their photosynthetic partner for their source of carbohydrates, lichenized fungi are characterized by their slow growth and their adaptation to low-nutrient habitats and sometime harsh abiotic conditions^3,4^. They are also known to produce high number of secondary metabolites^5,6^, which can accumulate in their thallus at high concentrations, up to 10–30% of their dry weight^7–9^. To date, more than 1,000 lichen compounds have been characterized, most of which are only found in lichens^9–11^. These compounds are biosynthesized by the fungal partner through three main biochemical pathways^7,9,11,12^: the polyketide (also known as acetyl-polymalonyl) pathway (eg, depsides, depsidones, dibenzofurans, anthraquinones, xanthones), the mevalonic acid pathway (terpenoids and steroids), and the shikimic acid pathway (eg, terphenylquinones, pulvinic acids). Although the functions of these compounds are still poorly understood^10,13^, they are generally thought to protect lichens from biotic (herbivory, pathogens) or abiotic (UV irradiations, desiccation) stresses. Due to their unique chemistry, lichens have long been used in traditional medicine, as well as in dyes and in the perfume industry^14^. As a result, the pharmaceutical properties of lichen extracts or lichen compounds are actively studied, and some species have been shown to have antimicrobial and anticancer activities^15–18^.

Historically, these metabolic compounds have been used to describe and identify lichenized fungal species, following a classification method called chemotaxonomy or chemosystematics^19,20^. As a result, the chemistry of lichens has been relatively well-studied as detected compounds are reported in most new species descriptions. Various methods have been available for both the elucidation of the structure of lichen compounds and their subsequent identification within a lichen thallus for species identification purpose. For elucidating the structure of lichen compounds, chemists have traditionally used ^13^C and ^1^H NMR spectroscopy, mass spectrometry and X-ray crystallography^21–24^. For species identification, lichen taxonomists have used spot tests (mostly using calcium hypochlorite and potassium hydroxide solutions)^12^, thin layer chromatography (TLC)^25,26^, and high-performance liquid chromatography (HPLC) to identify the lichen metabolite profiles^27,28^. Due to its accessibility and broad applicability, TLC rapidly became the most used method among lichen taxonomists^24^. However, this method has limitations, including the inability to detect low concentration compounds, poor resolving power and the dependency on the availability of standards for compound characterization^29^. As a result, more powerful MS/MS-based methods have been increasingly applied to detect secondary compounds in lichens^30–32^, and a new database of MS/MS spectra acquired by Q-TOF has been developed to allow for the identification of the compounds detected^29^. This Global Natural Product Social Molecular Networking (GNPS)-based database termed the Lichen Database (LDB) included 251 pure compounds isolated by the lichen chemist Siegfried Huneck and kept at the B Herbarium (Berlin, Germany) and by a research laboratory in Rennes (France). As such online MS/MS spectral library can be easily accessed by others, it has been subsequently used to detect secondary metabolites in several studies on lichens^32–35^.

To complement the previous work of Olivier-Jimenez *et al*.^29^, we are here contributing the MS/MS spectral data for 534 compounds isolated from lichens by Jack Elix as part of his five decade-long work on Australian lichens^24^. This collection of lichen compounds, which is subsequently referred to as ElixDB, includes the MS/MS spectra collected by Orbitrap of 534 compounds that have been uploaded to MetaboLights databases and also made available on a GNPS platform for molecular network analysis. This includes the MS/MS spectra for an additional 399 unique compounds, which takes the combined total to 650 out of the 1,000 known lichen compounds, now available in the MetaboLights and GNPS platforms, representing a more than doubling of the data available previously. The goal of this study is twofold: contribute further open-access spectral data of lichen compounds to: 1) allow refining the chemotaxonomy framework for lichens and; 2) increase the coverage of lichen compounds in existing spectral natural products libraries. These data will therefore be of benefit to both lichen taxonomists and systematists and to natural product chemists in facilitating dereplication in future lichen compound discovery studies.

## Methods

### Compound collection and sample preparation

The compounds were sourced from the collection of Jack Elix, containing over 50 years of natural isolates and semi-synthetic compounds derived from lichens. These compounds are currently hosted at the CANB herbarium (Canberra, Australia) and stored dry in glass containers at room temperature. Subsamples of each pure compound were taken with clean metal spatulas, transferred to Eppendorf tubes and weighed with an ABT-22-4M (Kern) precision balance for a target amount of 0.1–1 mg. For compounds with little material available, 50 µL of LCMS-grade MeOH were added to the glass container and 25 µl were pipetted back into an Eppendorf tube after mixing. Both the glass container and the Eppendorf tubes were then let to evaporate in a fume hood to dry the samples. All samples were reconstituted to 1 mg/mL in LCMS-grade MeOH. They were then sonicated, centrifuged and the supernatant was transferred to 1.5 mL LCMS vials for analysis.

### MS/MS data acquisition

A 1 µL sample was injected and analyzed using a Thermo Orbitrap Exploris 120 mass spectrometer equipped with an Electrospray ion Source (ESI) and a Thermo Vanquish ultra-high performance liquid chromatography (UHPLC) system (Thermo Fisher Scientific, USA). The column used was an Agilent InfinityLab Poroshell C18 (100 × 2.1 mm, 2.7 µm particle size) with mobile phase A being 0.1% FA Milli-Q H_2_O and mobile phase B being 0.1% FA in ACN. Following 1 min at 5% B, a gradient from 5% B to 95% B from over 3 min was employed for elution, followed by 100% B for 2 min and then re-equilibration at 5% B for 4 mins, all with a flow rate of 0.3 mL/min. A blank MeOH sample was run at the beginning of every batch and every ten samples otherwise. Polarity switching was utilized for each run, collecting data in both positive and negative for the first scan event, followed by four data-dependent MS/MS scans of the four most intense ions in the previous scan. Ions were excluded following selection for the next 4 sec. For all ions, merged spectra were generated from a combination of individual collision events at 10, 35 and 80 eV utilizing an isolation window of *m/z* 1.4. Internal mass calibration used Thermo EASY-IC. A scan range of *m/z* 140 – 2000 was utilized with an orbitrap resolution of 60,000 for MS events and 15,000 for MS/MS events. The ESI capillary voltage was set at 3500 V with a vaporizer temp of 350 °C and an ion transfer tube temp of 325 °C.

### Data analysis and database generation

Spectra were generated for all but 40 of the 574 analysed compounds (Table 1). A spreadsheet was generated with all of the compound names, molecular formula, accurate masses coupled to the raw format data file name. Utilizing Thermo Freestyle software, the corresponding M + H or M−H ion merged collision energy MS/MS scan was visualized for quality, fragment ions were identified for validation and then the scan exported to an individual raw file, which were all then converted to mzXML files using the MSConvert module from Proteowizard^36,37^. The files, along with their associated metadata, where then batch uploaded to MetaboLights as project ID MTBLS8109^38^, as well as GNPS (CCMSLIB00012718462 - CCMSLIB00012718995) where it was converted to a MS/MS library database titled “ELIXDB Lichen Database” for molecular network analysis (https://gnps.ucsd.edu/ProteoSAFe/gnpslibrary.jsp?library=ELIXDB-LICHEN-DATABASE)^39^. The database spectra were then manually checked and validated with test queries using molecular networking.

**Table 1:** Compounds that were provided by Jack Elix, analyzed and did not result in detectable molecular ions.

1	3β-Acetoxyfern-9(11)-ene-12β-ol	21	16β, 22-Dihydroxyhopan-4α-oic acid [Leucotylic acid]
2	3β-Acetoxyfern-9(11)-ene-19β-ol	22	Hierridin
3	3β-Acetoxyhopane-1β,22-diol	23	Hopane-6α,22-diol [Zeorin]
4	6α-Acetoxyhopane-16β,22-diol	24	Hopane-7β,22-diol
5	16β-Acetoxyhopane-6α,22-diol	25	Hyperpicrolichenic acid
6	20α-Acetoxyhopane-6α,22-diol	26	Isohyperplanaic acid
7	6α-Acetoxyhopane-22-ol [Acetylzeorin, Lesdainin]	27	Jackinic acid
8	7β-Acetoxyhopan-22-ol [Peltidactylin]	28	Letrouitic acid
9	15α-Acetoxyhopane-22-ol [Dolichorrizin]	29	Lupeol
10	Acetyl-α-tocopherol [Tocopheryl acetate; vitamin E acetate]	30	3-*O*-Methyldiploicin [4-*O*-Methyldiploicin]
11	Anhydrofusarubin lactol	31	2′-*O*-Methylhiascic acid
12	Anhydrofusarubin lactol methyl ketal	32	4-*O*-Methyloxocryptochlorophaeic acid
13	Butlerin C	33	8-O-Methylthiomelin
14	Butlerin E	34	Micareic acid
15	Butlerin F	35	Scabrosin diacetate [4,4′-Diacetylscabrosin]
16	7-Chlorocitreorosein	36	Strepsilin
17	7-Chloro-1,6,8-trihydroxy-3-methyl-9-anthrone	37	Superlatolic acid [Prasinic acid]
18	α-Collatolic acid	38	Tenuiorin
19	Coneuplectin	39	Wrightiin
20	Decarboxyalectoronic acid	40	Crustinic acid

This is possibly due to the compound not ionizing under ESI conditions, or the compound having broken down over years of storage.

### Database description and use as a metabolomic identification tool

**Fig. 1:**
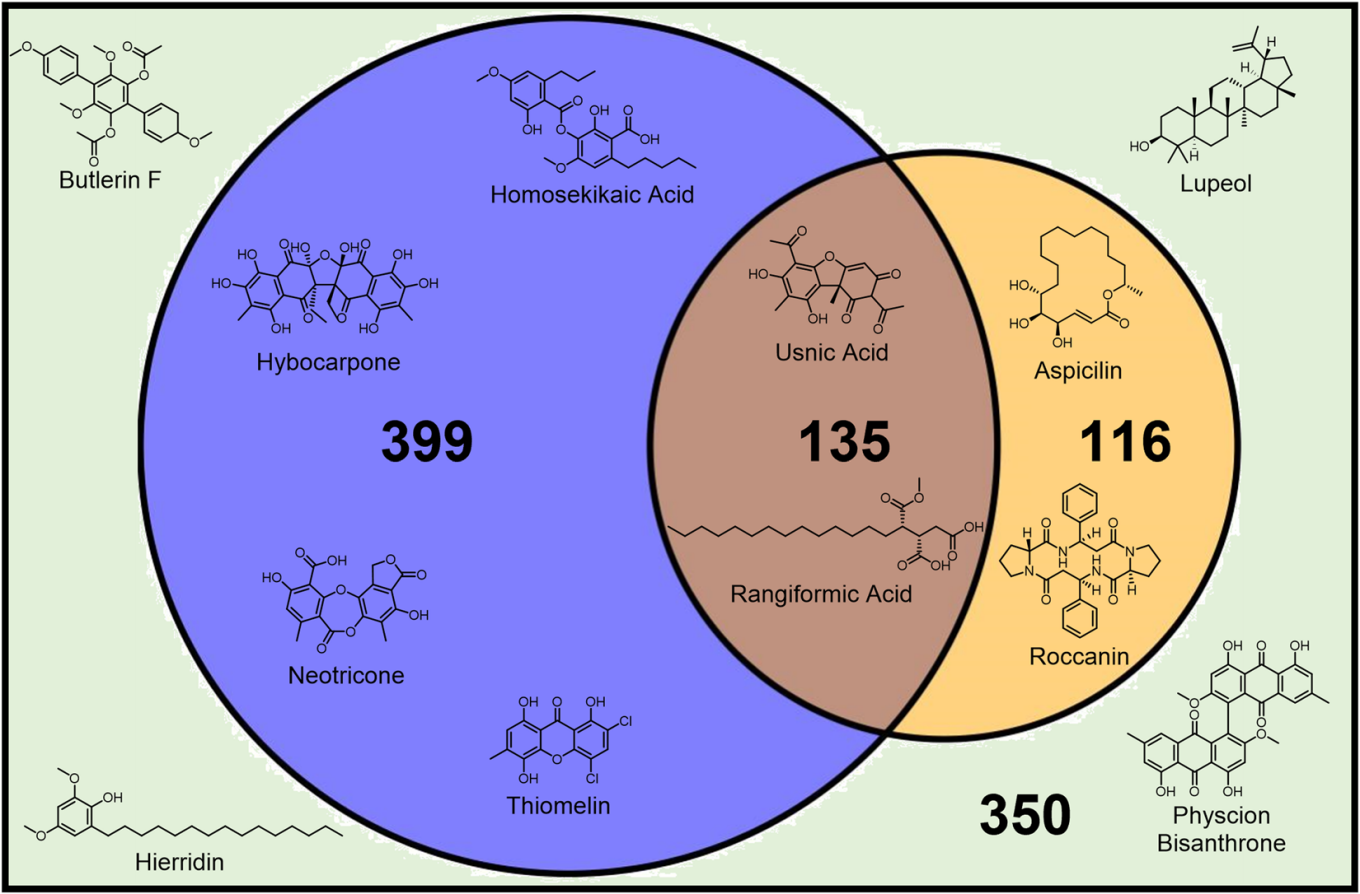
Venn diagram representing the proportion of lichen natural products for which MS/MS data is available, both in GNPS and MetaboLights. The purple area represents compounds unique to the newly generated ElixDB, the orange area compounds unique to LDB^29^ and the brown area compounds present in both. The green box represents the total number of reported lichen compounds.

**Fig. 2:**
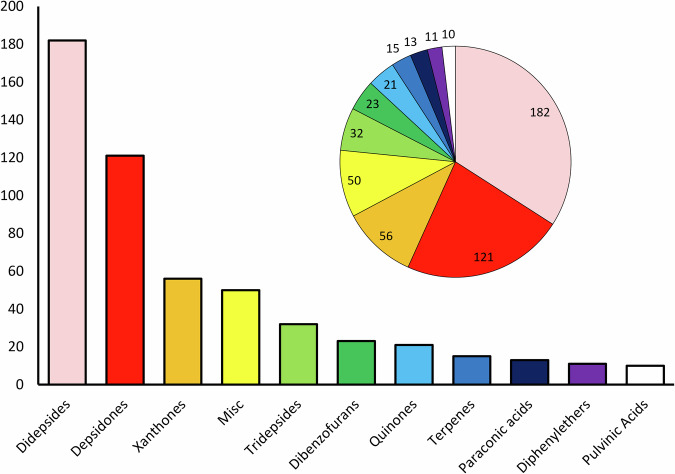
Proportion of lichen compound classifications uploaded as a part of the ElixDB.

Our database is to be used in conjunction with the LDB, both of which can be conveniently queried simultaneously using the GNPS online platform. Our database comprises the MS/MS spectra of 534 compounds, 399 of which were not present in the LDB (Fig. 1), taking the total number of lichen compounds from these two databases alone to 650. We utilized ESI for all compound ionization, as the goal of our database was to provide a convenient source of data for researchers interested in lichen taxonomy. ESI is a much more widely adopted ionization source than others such as APCI or EI due to its wider range of compound ionization, and we envisaged that LC-ESI-MS could potentially be used as a standalone tool to determine lichen taxonomy. The compounds in our database represent a wide range of structural classifications, with examples from all of the major lichen natural product classes including depsides, depsidones, xanthones and terpenoids (Fig. 2).

For lichen compound identification, researchers can extract a small portion of lichen in a solvent (typically MeOH or acetone for at least 1 h), filter, dry, resuspend in MeOH, centrifuge and inject 1 µL using the same data-dependent acquisition MS parameters above. Following conversion to the mzXML format using MSConvert, the extract file can then be uploaded to GNPS, with our database and the LDB selected for the ‘*Library Search’* experiment. This returns a list of all of the hit compounds in the sample, which can then be used to compare to known compounds of a species for taxonomic purposes. To explore the molecular diversity and probe for related compounds absent from our databases, users can select the ‘*Molecular Networking’* experiment which organizes nodes based on MS/MS spectral similarity. To this end, clusters of classes of compounds in the sample can be explored, as well as unidentified nodes connected to identified compounds which may represent novel chemistry within the organism.

## Data Records

The MS/MS merged spectra for all 534 compounds are present as mzXML data files. These can be accessed at MetaboLights as project ID MTBLS8109^39^ and also as a database on the GNPS platform^40^. These also contain metadata associated to each compound, including the exact mass, SMILES code and structural class.

## Technical Validation

The structures of all individual compounds were validated through various methods such as NMR, MS and TLC-comparisons at the time of isolation. The MS/MS spectra were run on the pure compounds, and the dominant fragment ions of each were individually analyzed to ensure they were consistent with the compound structure.

**Fig. 3:**
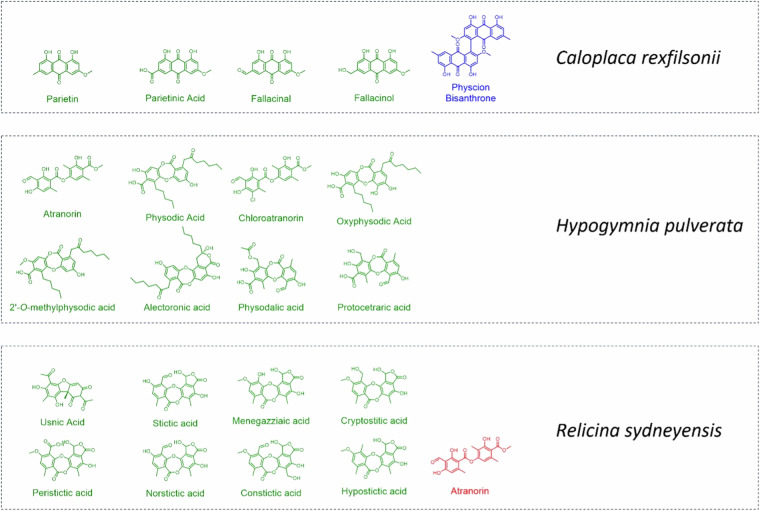
Compounds previously identified from the three Australian lichen sample extracts. The molecules are colored by the result of the GNPS library search. Green represents a ‘hit’, red represents not detected and the blue compound was not present in either database.

In order to validate our ElixDB^39,40^ working in conjunction with the LDB to identify metabolites based on MS/MS spectra, we extracted the thalli of three lichens specimens, *Hypogymnia pulverata* (CBG9608489), *Relicina sydneyensis* (CANB760164) and *Caloplaca rexfilsonii* (CANB774580), representing three Australian species. The acetone extract was processed as above, and 1 µL was run through the untargeted MS/MS protocol and uploaded to GNPS. The *Library Search* was used with a cosine score threshold of 0.7 and 6 minimum matched peaks. The results were then downloaded, analysed and compared to the chemistry expected based on the lichen literature and the annotations of the specimens, with the results summarized in Fig. 3 and Table 2.

**Table 2:** Number of compounds identified from extracts of three Australian lichens using the ElixDB and/or the LDB.

	ElixDB	LDB	Both	Missed	Total
*Caloplaca rexfilsonii* (CANB777480)	13	7	5	7	15
*Hypogymnia pulverata* (CBG9608489)	20	8	6	5	22
*Relicina sydneyensis* (CANB760164)	25	11	8	6	28

*Both* represents the number of compounds that were identified in both databases concurrently, *Missed* represents the number of compounds that were only identified by one database despite being present in both.

Of the 22 compounds that were previously associated with these lichen samples, 20 were identified using this database searching, including all eight compounds from *H. pulverata*. Of the two compounds not identified, physcoin anthrone has been recorded from *C. rexfilsonii* but is not present in either MS/MS database, whereas atranorin is present in both databases but was only present in trace quantities of the *R. sydneyensis* extract and wasn’t selected for fragmentation based on its low abundance below the threshold of data-dependent acquisition. In all lichen samples tested, many more compounds from our MS/MS databases were detected than expected based on previous reports, which was expected due to the vastly improved sensitivity and accuracy associated with MS techniques compared to traditional isolation and TLC methods. Prior to this experiment, these three lichens were attributed 22 different compounds based on the literature; however, we tentatively detected and matched a total of 65 compounds (Supplementary information document 1). Although it would require further verification if the unreported compounds were originated from tiny fragments of co-occurring lichen species sampled together with the target species, it nonetheless demonstrates the unprecedented power of lichen metabolite detection using MS/MS spectral search with the ElixDB.

During the analysis we noted that many compounds expectedly hit both databases, however several compounds hit only one database when they were present in both. For example, all three lichen samples returned a hit for caperatic acid in the ElixDB database, however only *C. rexfilsonii* returned a hit for the same compound in LDB. Whereas the norstitic acid hit was detected in *R. sydneyensis* with both databases but in *C. rexfilsonii*, the hit was only with ElixDB. While atranorin and physodalic acid are present in both databases, the two compounds only produced hits in ElixDB for *H. pulverata*. When the MS/MS spectra of these compounds uploaded to GNPS were manually interrogated, no problems were observed for either database and the fragment ions for both were as expected. Therefore, we take this opportunity to advise as a word of caution that, if a compound doesn’t hit the library, it doesn’t mean that it is absent from the lichen extract. MS is a technique that is sensitive to the instrument used and its running parameters, particularly when different collision energies are used between different users. Of the 18 total database ‘misses’ (compounds identified by one database and not the other despite being present in both) across the three samples, 17 missed the LDB library demonstrating a clear bias and the importance of replicating the running conditions of the database standards to achieve optimal dereplication. It is widely recognised that different types of instruments (Q-TOF, Orbitrap etc.) can lead to different fragmentation patterns and mechanisms^41^, therefore lichen extracts dereplicated by Q-TOF may prove to have a higher annotation rate for the LDB. This also demonstrates the power of searches when combining both databases, promoting this application for users with both types of instruments.

**Fig. 4:**
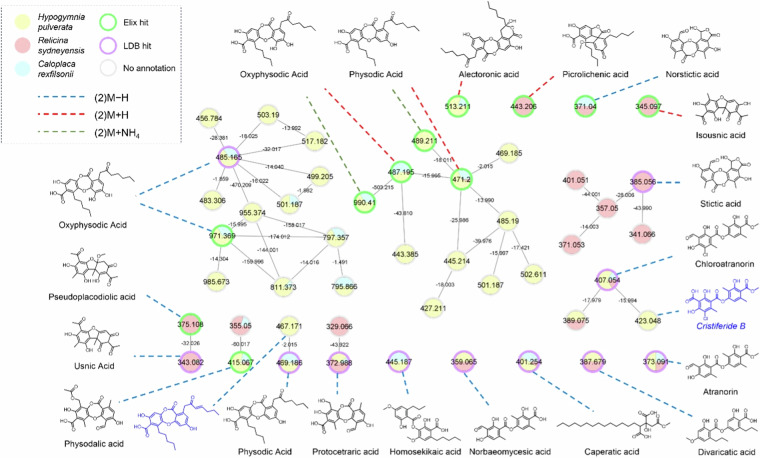
GNPS molecule network of the three Australian lichen samples and the identified metabolites in black, and postulated metabolites in blue. Nodes are labelled with precursor ion mass and edges are labelled with mass difference. Nodes are colored based on extract sample, and the boarder color is dependent on the source of annotation (if any). Color of the dotted line represents the ion type.

We next produced a combined molecular network of the three lichen species, utilizing the common GNPS settings of a 0.7 minimum pair cosine score and six minimum matched fragment ions for node connection (Fig. 4). Several identified metabolites were involved in the networking, including those from both databases. The LDB annotated 11 nodes, representing seven unique metabolites as several, such as physodic acid and oxyphysodic acid, had multiple types of ions (e.g M + H, M−H and M + NH_4_). Our database on the other hand, also annotated 11 nodes but represented 11 different compounds as only one ion per metabolite was uploaded.

The network of identified nodes consisted of two major clusters (one for negative ions and one for positive) related to oxyphysodic acid and physodic acid. Smaller clusters related to stictic acid and chloroatranorin were also detected, while the remaining clusters consisted of just one or two nodes. The identified compounds in the network were mainly didepsides and depsidones, possibly due to the large numbers of both in the databases and also their ease of fragmentation into similar daughter ions, favoring spectral alignment. The vast majority of identified compounds from the extracts from the Library Search were not present in the network, therefore molecular networking should not be the choice of experiment if dereplication is intended. The network does however, present several unidentified nodes (grey boarder) that may represent targets for novel molecule discovery. For example, the node labelled 467.171 is currently unidentified but possibly represents a physodic acid congener with a double bond in the lipid side chain as the ion is shifted by 2 mass units which typically indicates a further degree of unsaturation, and the node labelled 423.048 linked to chloroatranorin likely represents cristiferide B based on manual fragment ion analysis^42^. Cristiferide B was recently reported in 2019 and is not present in either of the uploaded databases. Through both the Library Search and Molecular Networking on the GNPS platform, we were able to clearly observe robust integration of our database to be used in conjunction with the LDB. With a combined composition of over 650 secondary metabolites, complementary use of both databases resulted in the dereplication of over 90% of the previously reported metabolites across the three lichen species *H. pulverata*, *R. sydneyensis* and *C. rexfilsonii*. This outcome supports the technical validation of our database. The sensitivity and high-resolution accuracy of this technique also led to the detection of many other previously unassociated compounds, that were either identified through the database or could have their structures tentatively proposed based on their associated clusters using molecular networking. Additional analyses (ie, HPLC) would be necessary to confirm whether these additional compounds belong to the target species or contaminating fragments of co-occurring species.

We believe that the significantly expanded library of lichen compound MS/MS spectra with ElixDB will contribute to both lichen systematics and compound discovery. The power of MS/MS-based methods for lichen compound detection will continue to be improved with more MS spectra being uploaded to expand the databases of lichen compounds in the future.

## Supplementary information

Supplementary information document 1

## Data Availability

No custom code was used.

## References

[1] Galloway, D. J. Biodiversity: a lichenological perspective. *Biodivers. Conserv.* **1**, 312–323 (1992).

[2] Kirk, P.M., Ainsworth, G.C., Bisby, G.R. & International, C.A.B. *Ainsworth & Bisby’s Dictionary Of The Fungi*, Edn. 10th. (CAB International, Wallingford, United Kingdom; 2008).

[3] Beckett, R.P., Kranner, I. & Minibayeva, F.V. Stress physiology And The symbiosis, in *Lichen Biology*, Edn. 2nd edition 134–151 (Cambridge University Press, Cambridge; 2008).

[4] Kranner, I., Beckett, R. P., Hochman, A. & Nash, T. H. Desiccation-Tolerance in Lichens: A Review. *Bryologist* **111**, 576–593 (2008).

[5] Asahina, Y. & Shibata, S. *Chemistry Of Lichen Substances*. (Japan Society for the Promotion of Science, Ueno, Tokyo; 1954).

[6] Huneck, S. & Yoshimura, I. *Identification Of Lichen Substances*. (Springer Berlin Heidelberg, Berlin, Heidelberg; 1996).

[7] Galun, M. & Shomer-Ilan, A. Secondary metabolic products, in *Handbook of Lichenology*, Vol. III, Edn. 1st Edition 3–8 (CRC Press, Boca Raton; 1988).

[8] Huneck, S. Chapter 15 - Nature of lichen substances, in *The Lichens*. (eds. V. Ahmadjian & M. E. Hale) 495–522 (Academic Press, New York and London; 1973).

[9] Stocker-Wörgötter, E. Metabolic diversity of lichen-forming ascomycetous fungi: culturing, polyketide and shikimatemetabolite production, and PKS genes. *Nat. Prod. Rep.* **25**, 188–200 (2008). 18250902 10.1039/b606983p

[10] Calcott, M. J., Ackerley, D. F., Knight, A., Keyzers, R. A. & Owen, J. G. Secondary metabolism in the lichen symbiosis. *Chem. Soc. Rev.* **47**, 1730–1760 (2018). 29094129 10.1039/c7cs00431a

[11] Goga, M. *et al*. Lichen Metabolites: An Overview of Some Secondary Metabolites and Their Biological Potential, in *Co-Evolution of Secondary Metabolites*. (eds. J.-M. Mérillon & K.G. Ramawat) 175–209 (Springer International Publishing, Cham; 2020).

[12] Culberson, C. F. & Elix, J. A. Lichen Substances, in *Methods in Plant Biochemistry*, Vol. 1. (ed. J. B. Harborne) 509–535 (Academic Press, London and San Diego; 1989).

[13] Molnár, K. & Farkas, E. Current Results on Biological Activities of Lichen Secondary Metabolites: a Review. *Z. Naturforsch. C J. Biosci.* **65**, 157–173 (2010). 20469633 10.1515/znc-2010-3-401

[14] Elix, J.A. & Stocker-Wörgötter, E. Biochemistry And Secondary Metabolites, in *Lichen Biology*, Edn. 2nd edition 104–133 (Cambridge University Press, Cambridge; 2008).

[15] Maurya, I. K. *et al*. Antimicrobial activity of Bulbothrix setschwanensis (Zahlbr.) Hale lichen by cell wall disruption of Staphylococcus aureus and Cryptococcus neoformans. *Microb. Pathog.* **115**, 12–18 (2018). 29223452 10.1016/j.micpath.2017.12.015

[16] Resende, D. I. S. P. *et al*. Lichen Xanthones as Models for New Antifungal Agents. *Molecules* **23**, 2617 (2018). 30322037 10.3390/molecules23102617 PMC6222623

[17] Sahoo, B., Dash, S., Parida, S., Sahu, J. K. & Rath, B. Antimicrobial activity of the lichens Parmotrema andium and Dirinaria applanata. *J. Appl. Biol. Biotechnol.* **9**, 93–97 (2021).

[18] Gautam, A. K., Yadav, D., Singh, P. K., Bhagyawant, S. S. & Jin, J. O. Lichen: A comprehensive review on Lichens as a natural sources exploring nutritional and biopharmaceutical benefits. *Prog. Nutr.* **23**, e2021153 (2021).

[19] Culberson, W. L. Chemosystematics and Ecology of Lichen-Forming Fungi. *Ann. Rev. Ecol. & Syst.* **1**, 153–170 (1970).

[20] Feige, G.B., Lumbsch, H.T. & Huneck, S. *Phytochemistry And Chemotaxonomy Of Lichenized Ascomycetes: A Festschrift In Honour Of Siegfried Huneck, Volume 53 Of Bibliotheca lichenologica*. (J. Cramer, 1993).

[21] Aubert, S., Juge, C., Boisson, A., Gout, E. & Bligny, R. Metabolic processes sustaining the reviviscence of lichen Xanthoria elegans (Link) in high mountain environments. *Planta* **226**, 1287–1297 (2007). 17574473 10.1007/s00425-007-0563-6 PMC2386907

[22] Eisenreich, W., Knispel, N. & Beck, A. Advanced methods for the study of the chemistry and the metabolism of lichens. *Phytochem. Rev.* **10**, 445–456 (2011).

[23] Xu, B., Sung, C. & Han, B. Crystal Structure Characterization of Natural Allantoin from Edible Lichen Umbilicaria esculenta. *Crystals* **1**, 128–135 (2011).

[24] Elix, J. A. *A Catalogue Of Standardized Chromatographic Data And Biosynthetic Relationships For Lichen Substances*, Edn. 6th. (The author, Canberra; 2022).

[25] Culberson, C. F. Improved conditions and new data for identification of lichen products by standardized thin-layer chromatographic method. *J Chromatogr. A.* **72**, 113–125 (1972). 10.1016/0021-9673(72)80013-x 5072880

[26] Orange, A., James, P.W. & White, F.J. *Microchemical Methods For The Identification Of Lichens*, Edn. 2nd edition. (British Lichen Society, London; 2010).

[27] Feige, G. B., Lumbsch, H. T., Huneck, S. & Elix, J. A. Identification of lichen substances by a standardized high-performance liquid chromatographic method. *J. Chromatogr. A* **646**, 417–427 (1993).

[28] Elix, J. A., Giralt, M. & Wardlaw, J. H. New chloro-depsides from the lichen Dimelaena radiata. *Bibliotheca Lichenologica* **86**, 1–7 (2003).

[29] Olivier-Jimenez, D. *et al*. A database of high-resolution MS/MS spectra for lichen metabolites. *Scientific Data* **6**, 294 (2019). 31780665 10.1038/s41597-019-0305-1 PMC6882832

[30] Kumar, K. *et al*. UPLC–MS/MS quantitative analysis and structural fragmentation study of five Parmotrema lichens from the Eastern Ghats. *J. Pharm. Biomed. Anal.* **156**, 45–57 (2018). 29689468 10.1016/j.jpba.2018.04.017

[31] Sveshnikova, N., Yuan, T., Warren, J. M. & Piercey-Normore, M. D. Development and validation of a reliable LC–MS/MS method for quantitative analysis of usnic acid in Cladonia uncialis. *BMC Res. Notes* **12**, 550 (2019). 31470895 10.1186/s13104-019-4580-x PMC6716858

[32] Lagarde, A. *et al*. Chlorinated bianthrones from the cyanolichen Nephroma laevigatum. *Fitoterapia* **149**, 104811 (2021). 33359429 10.1016/j.fitote.2020.104811

[33] Condò, C. *et al*. Lichens as a Natural Source of Compounds Active on Microorganisms of Human Health Interest. *Appl. Sci.* **13**, 1976 (2023).

[34] Vaez, M. & Javad Davarpanah, S. New Insights into the Biological Activity of Lichens: Bioavailable Secondary Metabolites of Umbilicaria decussata as Potential Anticoagulants. *Chem. Biodivers.* **18**, e2100080 (2021). 33773025 10.1002/cbdv.202100080

[35] Norouzi, H., Azizi, A., Gholami, M., Sohrabi, M. & Boustie, J. Chemotype variations among lichen ecotypes of Umbilicaria aprina as revealed by LC-ESI-MS/MS: a survey of antioxidant phenolics. *Environ. Sci. Pollut. Res.* **27**, 40296–40308 (2020). 10.1007/s11356-020-10053-2 32661964

[36] Adusumilli, R. & Mallick, P. Data Conversion with ProteoWizard msConvert, in *Proteomics: Methods and Protocols*. (eds. L. Comai, J.E. Katz & P. Mallick) 339–368 (Springer New York, New York, NY; 2017). 10.1007/978-1-4939-6747-6_23 28188540

[37] Chambers, M. C. *et al*. A cross-platform toolkit for mass spectrometry and proteomics. *Nat. Biotech.* **30**, 918–920 (2012). 10.1038/nbt.2377 PMC3471674 23051804

[38] Wang, M. *et al*. Sharing and community curation of mass spectrometry data with Global Natural Products Social Molecular Networking. *Nat. Biotechnol.* **34**, 828–837 (2016). 27504778 10.1038/nbt.3597 PMC5321674

[39] Bracegirdle, J. *et al*. MTBLS8109: An Expanded Database of High-Resolution MS/MS Spectra for Lichen Derived Natural Products. *Metabolights* https://identifiers.org/metabolights:MTBLS8109 (2024). 10.1038/s41597-025-04488-w PMC11814408 39934125

[40] Bracegirdle, J. *et al*. Elix Lichen Compounds V02. *GNPS* https://gnps.ucsd.edu/ProteoSAFe/gnpslibrary.jsp?library=ELIXDB-LICHEN-DATABASE (2024).

[41] Cao, X., Cai, X. & Mo, W. Comparing the fragmentation reactions of protonated cyclic indolyl α-amino esters in quadrupole/orbitrap and quadrupole time-of-flight mass spectrometers. *Rapid Commun. Mass Spectrom.* **32**, 543–551 (2018). 29369433 10.1002/rcm.8063

[42] Pham, N.-K.-T. *et al*. Two new phenolic compounds from the lichen Parmotrema cristiferum growing in Vietnam. *Nat. Prod. Res.* **36**, 3865–3871 (2022). 33656403 10.1080/14786419.2021.1892672

