# Supplementary material for: An expanded database of high-resolution MS/MS spectra for lichen-derived natural products
Source: Sci Data. 2025 Feb 12;12:244. doi: 10.1038/s41597-025-04488-w (PMC11814408; doi:10.1038/s41597-025-04488-w)
Supplement: Supplementary file 1 — Supplementary information document 1 [file 41597_2025_4488_MOESM1_ESM.docx]

Supplementary information document 1. Compounds dereplicated from test lichens using the ElixDB and LDB databases on GNPS. Compounds in bold are reported from the species for the first time.

| **Organism** | **Compound Name** | **Database** |
| --- | --- | --- |
| *Hypogymnia pulverata* | 2-*O*-Methylphysodic acid | ElixDB |
|  | **9-Methylprotocetraric acid** | LDB |
|  | alpha-Alectoronic acid | LDB + ElixDB |
|  | Atranorin | ElixDB |
|  | beta-Alectoronic acid | ElixDB |
|  | **Caperatic acid** | LDB + ElixDB |
|  | Chloroatranorin | ElixDB |
|  | **Constipatic acid** | ElixDB |
|  | **Divaricatic acid** | ElixDB |
|  | **Glomelliferonic acid** | ElixDB |
|  | **Haematommic acid** | LDB |
|  | **Homosekikaic acid** | ElixDB |
|  | **Methyl beta-orsellinate or Atratic acid** | ElixDB |
|  | **Methyl pseudoalectoronate** | ElixDB |
|  | **Norbaeomycesic acid** | ElixDB |
|  | Oxyphysodic acid | LDB + ElixDB |
|  | Physodalic acid | ElixDB |
|  | Physodic acid | LDB + ElixDB |
|  | Protocetraric acid | LDB + ElixDB |
|  | **Thamnolic acid** | ElixDB |
|  | **Thiomelin** | ElixDB |
|  | **Usnic acid** | LDB + ElixDB |
|  |  |  |
| *Relicina sydneyensis* | **4-*O*-Methylhypoprotocetraric acid** | ElixDB |
|  | **Caperatic acid** | ElixDB |
|  | **Connorstictic acid** | ElixDB |
|  | Constictic acid | LDB + ElixDB |
|  | Cryptostictic acid | ElixDB |
|  | **Hyposalazinic acid** | ElixDB |
|  | Hypostictic acid | ElixDB |
|  | **Isousnic acid** | LDB |
|  | Menegazziaic acid | ElixDB |
|  | **Methyl pseudonorstictate** | ElixDB |
|  | **Methyl stictic acid** | ElixDB |
|  | **Neotricone** | ElixDB |
|  | Norstictic acid | LDB + ElixDB |
|  | **Notatic acid** | LDB + ElixDB |
|  | **Oxyphysodic acid** | LDB + ElixDB |
|  | **Pannaric acid** | ElixDB |
|  | Peristictic acid | ElixDB |
|  | **Physodalic acid** | LDB |
|  | **Physodic acid** | LDB + ElixDB |
|  | **Picrolichenic acid** | LDB + ElixDB |
|  | **Rangiformic acid** | LDB |
|  | **Salazinic acid** | ElixDB |
|  | **Scrobiculin** | LDB |
|  | Stictic acid | LDB + ElixDB |
|  | **Subnorstictic acid** | ElixDB |
|  | **Thiomelin** | ElixDB |
|  | Usnic acid | LDB + ElixDB |
|  | **Virensic acid** | ElixDB |
|  |  |  |
| *Caloplaca rexfilsonii* | **alpha-Alectoronic acid** | LDB + ElixDB |
|  | **Caperatic acid** | ElixDB |
|  | **Chloroatranorin** | ElixDB |
|  | **Emodic acid** | LDB |
|  | Fallacinal | LDB |
|  | Fallacinol (Teloschistin) | LDB + ElixDB |
|  | **Homosekikaic acid** | ElixDB |
|  | **Norstictic acid** | ElixDB |
|  | **Oxyphysodic acid** | LDB + ElixDB |
|  | Parietin | ElixDB |
|  | Parietinic acid | ElixDB |
|  | **Physodalic acid** | ElixDB |
|  | **Physodic acid** | LDB + ElixDB |
|  | **Protocetraric acid** | LDB + ElixDB |
|  | **Usnic acid** | ElixDB |
